# Characterization of two Pro-opiomelanocortin gene variants and their effects on carcass traits in beef cattle

**DOI:** 10.1186/1471-2156-12-2

**Published:** 2011-01-04

**Authors:** Heather M Deobald, Fiona C Buchanan

**Affiliations:** 1 Department of Animal and Poultry Science, University of Saskatchewan, Saskatoon, Canada; 2 Quantum Genetics Canada Inc., Saskatoon, SK Canada

## Abstract

**Background:**

Carcass quantity (lean meat yield) and quality (degree of marbling) in beef cattle determines much of their economic value. Consequently, it is important to study genes that are part of the appetite pathway and that may ultimately affect carcass composition. Pro-opiomelanocortin is a prohormone that codes for many different peptides, several of which are involved in the appetite pathway. A single nucleotide polymorphism (SNP) c.288C>T in pro-opiomelanocortin (*POMC*) has previously been associated with hot carcass weight (HCW) and shipping weight (Ship wt) in beef cattle.

**Results:**

While developing a commercial real time PCR test for *POMC *c.288C>T a 12 bp deletion *(POMC *c.293_304delTTGGGGGCGCGG) was identified. The deletion results in the removal of four amino acids (a valine, two glycines, and an alanine). Both the *POMC *c.288C>T and the deletion were genotyped in 386 crossbred steers and evaluated for associations with carcass traits. The animals with one copy of the deletion had a significantly smaller carcass rib-eye area (7.91 cm^2^; P = 0.02) in comparison to homozygous normal animals. Significant associations were observed between *POMC *c.288C>T with start-of-finishing weight (SOF WT; P = 0.04), hot carcass weight (HCW; P = 0.02), average fat and grade fat (both P = 0.05), carcass rib-eye area (REA; P = 0.03) and marbling (P = 0.02).

**Conclusions:**

These results suggest that it could be beneficial for beef producers to know both the deletion and *POMC *c.288C>T genotypes when making marketing and culling decisions.

## Background

The hypothalamus is a key area of the brain for appetite regulation. More specifically, there are two neuronal circuits, in the arcuate nucleus of the hypothalamus, that are important in both nutritional status and regulating energy homeostasis. One neuronal circuit contains the anorexigenic neuropeptides, including pro-opiomelanocortin (POMC), and the other circuit contains orexigenic peptides such as neuropeptide Y [1].

Pro-opiomelanocortin is a prohormone that codes for many different peptides including adrenocorticotrophin (ACTH), alpha melanocyte stimulating hormone (α-MSH), beta melanocyte stimulating hormone (β-MSH), gamma melanocyte stimulating hormone (γ-MSH), and β-endorphin (β-END) some of which are involved in the appetite pathway [2]. The main *POMC *encoded anorectic peptide responsible for reductions in food intake and appetite is αMSH which reduces appetite when bound to melanocortin 4-receptor (MC4R) or melanocortin 3-receptor (MC3R); γ and β MSH also reduce appetite but not to the same magnitude as α-MSH.

The prohormone POMC undergoes many post-translational cleavages as it passes through the Golgi bodies via the regulated secretory pathway (RSP) [3]. POMC is cleaved by prohormone convertases (PC1 and PC2) to produce the range of bioactive peptides [2,4,5]. The prohormone convertases (PC) cleave at the eight pairs, and one quadruplet, of basic amino acids contained within POMC [5].

The importance of *POMC *in the appetite pathway has made it a strong candidate gene for obesity in humans and carcass traits in livestock. Mutations in *POMC *in humans have been associated with obesity [6,7]. In beef cattle Thue and Buchanan [8] discovered *POMC *c.288C>T and mapped it to BTA 11: this SNP does not alter the amino acid - it remains a serine. This area is known to harbour a quantitative trait locus (QTL) affecting carcass weight and average daily gain [8]. Buchanan *et al. *[9] found an association between *POMC *c.288C>T with ship weight and hot carcass weight (HCW) in 256 Charolais cross steers.

The value of beef is currently determined based on lean meat yield and the extent of marbling. Consequently, it is important to study genes that are part of the appetite pathway and that may ultimately affect carcass composition. Since an association between *POMC *c.288C>T with hot carcass weight and shipping weight had already been established [9], it was important validate this association in another population.

## Results

### Discovery, characterization and association studies for the 12 bp deletion

**Figure 1:**
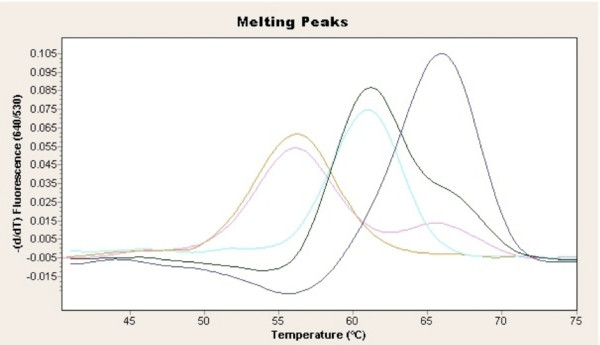
**Melting temperature peaks for the *POMC *c.288C>T real time PCR**. Tm at 61°C corresponds to the T allele, Tm at 66°C the C allele and Tm at 56°C represents an abnormal melting curve from the *POMC *c.288C>T genotyping assay

**Figure 2:**
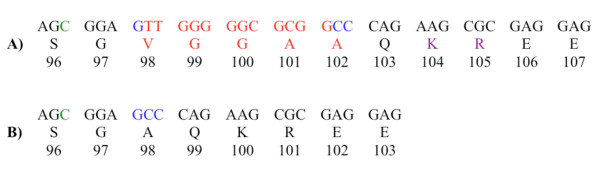
**Partial sequence from exon three of *POMC***. a) The 12 bp that are deleted and the affected amino acids in red, the first position in the valine codon and the 2nd and 3rd positions of the second alanine are coloured blue and *POMC *c.288C>T is coloured green. Purple represents the KR cleavage site b) the same sequence minus the 12 bps and 4 amino acids showing how the DNA and protein sequence comes back into alignment.

During optimization of a real time PCR assay for *POMC *c.288C<T, some cattle exhibited an additional melting curve at 56°C (Figure 1). Samples exhibiting the additional melting curve were sequenced and a 12 bp deletion was discovered - *POMC *c.293_304delTTGGGGGCGCGG [GenBank: GQ280285]. The deletion begins 5 bp downstream from *POMC *c.288C>T and results in the removal of 4 amino acids from the translated sequence (Figure 2). While the deletion starts in the second position of codon 98 (valine), the G from the first position combines with the CC from the second and third positions of codon 102 to recreate alanine bringing the amino acid sequence back into order. The amino acids removed are a valine, two glycines, and an alanine (Figure 2).

The primers used to amplify *POMC c.288C>T *were located within exon 3, therefore a series of PCRs were designed to rule out the possibility that the deletion and/or *POMC *c.288C>T was in a pseudogene. A set of five samples that were CT at *POMC *c.288C>T and heterozygous for the deletion were used for detection of a pseudogene. Using a forward primer located in intron 2, the genotype at both variants remained unchanged, thus ruling out the possibility of a processed pseudogene that are intronless. Using this intronic forward primer with a new reverse in the 3'UTR we tried to extend beyond any duplication of the gene; again the genotypes at the deletion and *POMC *c.288C>T did not change. Since the deletion occurs in close proximity to *POMC *c.288C>T, we wanted to confirm that both SNP alleles are expressed. From a panel of tissue samples, it was determined that *POMC *is transcribed in thymus. Thymus tissue was collected from five calves and genotyped for *POMC *c.288C>T using genomic DNA. Subsequently, cDNA was amplified using intron-spanning primers. Two of the calves were CT using genomic DNA and they remained CT when cDNA was the template.

To genotype for the deletion, PCR-amplified products were separated on a 4% agarose gel. The 386 steers were genotyped for the deletion. Fourteen animals were heterozygous for the deletion (+/-); thirteen of these were CT at *POMC *c.288C>T and one was homozygous TT (Table 1). The deletion was always associated with T allele of *POMC *c.288C>T (*i.e*. the deletion was never found with the C allele), however there were animals with the T allele that did not have the deletion. None of the samples tested were homozygous for the deletion; however, based on the low allele frequency of the deletion (0.02), this was not unexpected (our observed genotypes were in Hardy Weinberg Equilibrium (HWE)). We also genotyped five breeds of beef cattle plus Holsteins for the deletion (Table 1); a Chi squared analysis revealed there were no significant differences in the allele frequencies among breeds (P>0.05).

**Table 1:** Deletion genotypes and Minor Allele Frequency (MAF) of the deletion in six populations.

Population	n	+/+	+/-	-/-	MAF
Steers	386	372	14	0	0.02
Angus	50	46	4	0	0.04
Hereford	50	48	2	0	0.02
Simmental	50	48	2	0	0.02
Charolais	50	49	1	0	0.01
Limousin	50	48	2	0	0.02
Holstein	20	18	2	0	0.05

(+) denotes no deletion and (-) denotes the occurrence of the deletion.

Mann-Whitney U tests were performed to examine associations between *POMC *c.293_304delTTGGGGGCGCGG and carcass traits. Using only CT animals (n = 151), animals heterozygous for the deletion (n = 13; +/-) were compared to homozygous non-deletion animals (n = 138; +/+). A trend was observed for higher ultrasound EOB REA in animals that lacked the deletion at (P = 0.08). Animals lacking the deletion had a mean REA of 65.2 cm^2^, while animals heterozygous for the deletion had a mean REA of 60.9 cm^2^. The differences in REA are even more pronounced, in the carcass, at the end of finishing (P = 0.02) with values of 103.6 cm^2 ^and 95.7 cm^2 ^for homozygous and heterozygous animals respectively.

### Association studies with the *POMC *c.288C>T

The allele frequencies were 0.75 and 0.25 for the C and T allele, respectively. The Mixed procedure of SAS was used to determine if the *POMC *SNP genotype had an association with production or carcass traits (Table 2). The CC genotype was significantly lighter or smaller than the CT genotype for SOF WT, HCW and carcass REA and CC was significantly higher for two fat traits (Average fat and grade fat) than the CT or TT genotypes. For marbling Score, CC was significantly smaller than both CT and TT genotypes. Cutablility also increased numerically with the addition of each T allele, however it was only a statistical trend with a P-value of 0.06.

**Table 2:** Association analysis of carcass traits for 372 crossbred steers with POMC c.288C>T (LSM(±SEM))

Trait	CC (n = 210)	CT (n = 138)	TT (n = 24)	P-Value
SOB WT (kg)	243.0 ± 1.34	246.3 ± 1.66	242.2 ± 3.97	0.27
EOB WT (kg)	383.7 ± 2.06	389.8 ± 2.54	384.1 ± 6.10	0.18
B-ADG (kg/day)	1.2 ± 0.01	1.2 ± 0.02	1.2 ± 0.04	0.56
EOB BF (mm)	2.3 ± 0.10	2.2 ± 0.12	2.2 ± 0.28	0.74
EOB REA (cm^2^)	64.1 ± 0.52	65.2 ± 0.64	67.0 ± 1.53	0.12
SOF WT (kg)	426.6 ± 2.24^b^	435.5 ± 2.76^a^	427.6 ± 6.62^ab^	**0.04**
EOF WT (kg)	628.1 ± 3.75	639.6 ± 4.60	624.3 ± 10.98	0.12
F ADG (kg/day)	2.3 ± 0.02	2.3 ± 0.03	2.2 ± 0.07	0.45
HCW (kg)	373.6 ± 1.98^b^	382.5 ± 2.44^a^	376.9 ± 5.86^ab^	**0.02**
Average Fat (mm)	10.1 ± 0.25^a^	9.4 ± 0.31^ab^	8.5 ± 0.74^b^	**0.05**
Grade Fat (mm)	8.7 ± 0.25^a^	7.9 ± 0.31^b^	7.3 ± 0.74^ab^	**0.05**
Carcass REA (cm^2^)	100.4 ± 0.81^b^	103.6 ± 0.10^a^	104.0 ± 2.40^ab^	**0.03**
Marbling Score	7.7 ± 0.04^b^	7.8 ± 0.05^a^	7.9 ± 0.12^a^	**0.02**
Cutability (%)	60.8 ± 0.21	61.4 ± 0.27	62.2 ± 0.65	*0.06*

^ab ^Means in the same row with different superscripts are significantly different (P ≤ 0.05)

SOB WT = start of backgrounding weight, EOB WT = end of backgrounding weight, ,B-ADG = backgrounding average daily gain

EOB BF = end of backgrounding back fat, EOB REA = end of backgrounding, rib-eye area, SOF WT = start of finishing weight, EOF

WT = end of finishing weight, F ADG = finishing average daily gain, HCW = hot carcass weight

## Discussion

### Deletion

**Figure 3:**
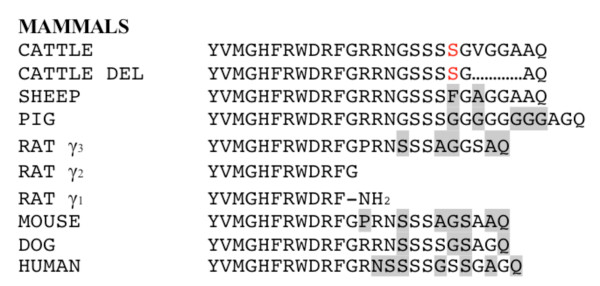
**Alignment of γ-MSH from various mammalian species**. Red color indicates position of *POMC *c.288C>T. Highlighting indicates variations from cattle sequence.

Sequence alignment using genomic DNA confirmed that the *POMC *c.293_304delTTGGGGGCGCGG deletion occurs in the γ-MSH peptide of cattle (Figure 3). In mammals the 5' end of the γ-MSH sequence is highly conserved but the 3' end is not. This lack of conservation is thought to be why the short forms of γ-MSH are still active in mice and rats [10,11]. While the 3' location of the deletion in γ-MSH likely does not affect its bioactivity, there may be a gene processing failure based on its proximity to the dibasic amino acids KR (Figure 1). These two amino acids immediately follow the γ-MSH peptide and are the recognition site for prohormone convertase 1 (PC1) to cleave the NH_2 _terminal peptide (containing γ-MSH) from the joining peptide in the corticotroph cells in the pituitary [4,5]. If this cleavage does not occur, the altered folding of the resultant longer peptide may also impact the cleavage of ACTH and subsequently α-MSH.

The POMC deletion sequence was analyzed using Predict Protein [12], and the program revealed the loss of a glycosaminoglycan (GAG) recognition site. GAG is predicted to bind to the amino acid sequence SGVG, but with the deletion the amino acid sequence instead becomes SGAQ. The loss of the GAG binding site could be important to the processing of POMC since GAGs are involved in the secretory pathway through the Golgi bodies [13]. This pathway is where various post-translational modifications occur, including prohormone convertase cleavage and compartmentalization for transport to the cell membrane [2]. Without the ability to bind GAG, POMC may not be able to properly pass through the Golgi bodies to be cleaved by prohormone convertases or the prohormone may not be transported to the cell membrane for secretion.

It can be postulated from the two mechanisms outlined above that there could be an effect on appetite and subsequently on production and/or carcass traits. Cattle heterozygous for the deletion had smaller Carcass REA by 7.9 cm^2 ^(8%). The *POMC *deletion was always found in conjunction with the T allele at the *POMC *c.288C>T SNP; however, 162 animals had the T allele (138 CT and 24 TT) without the deletion. This supports the theory that the deletion occurred after the SNP. No homozygous deletion individuals were found, but with the low allele frequency of the deletion this was not unexpected.

If the deletion significantly impairs POMC processing, animals homozygous for the deletion might display reduced viability. In mice and humans deficiency of POMC derived proteins has been shown to reduce viability [14,6]. It is not known to what extent the 12 bp deletion impacts the processing of POMC. If the deletion does result in low viability it would be important for breeders to select for cattle that do not possess the deletion. If viability is unaffected the gene test could still be useful to select for REA. Although the MAF is low it could be utilized to supply specialty markets that prefer smaller REA or improve efficiencies in large feedlots.

### *POMC *c.288C>T

The allele frequencies for *POMC *c.288C>T were 0.25 and 0.75 for the T and C allele, respectively; this is very similar to the allele frequencies reported by Buchanan et al. [9] in 256 Charolais-cross steers. The observed genotype frequencies were in HWE indicating that fitness is not being affected. Buchanan et al. [9] found a significant association between the SNP and shipping weight and HCW. This study has confirmed the association with HCW in a second, larger population of steers (n = 372). The T allele was associated with a significant increase of 8.89 kg in HCW between the CC and CT genotypes. This is very similar to the 7.4 kg increase in HCW observed between CC and CT by Buchanan et al. [9]. A significant association was also found between the SNP and carcass REA - the addition of one T allele was associated with an increase of 3.23 cm^2 ^in the REA. Thus, the increase in HCW is likely due to increased muscle mass, especially given the fact that average fat and grade fat were significantly decreased with the addition of each T allele. The increase in REA with a decrease in fat measurements makes for a consistent story since tissue and fat deposition are in opposition to one another. At first glance, the association with marbling seems contradictory as the directionality of the association is in the opposite direction to grade fat and average fat. Although the carcasses were graded on a 2-9 point scale only grades 7, 8 or 9 were found in this population. The LSMs reported while significant would not alter the marbling grade received in either the Canadian or US meat grading systems.

Although the *POMC *c.288C>T SNP is a silent mutation it could still have a significant effect on translation or transcription efficiency. For example, not all tRNAs are equally abundant, some tRNAs bind to more than one codon, exon splicing can be affected, folding of mRNA and G or C bases may increase mRNA synthesis [15,16]. The *POMC *c.288C>T is a C to T substitution and may result in a reduction in *POMC *expression. Consequently, there would be an increased appetite due to less α-MSH production. The findings of this study support this theory, as an increase in HCW was found with the addition of a T allele (Table 2).

## Conclusions

In summary we supported the originally finding of an association between *POMC *c.288C>T and HCW. In addition, we also found statistical associations with the SNP and carcass-REA, grade fat and average fat. This research also led to the discovery of a novel 12 bp deletion in *POMC*, and further analysis of the deletion showed a significant correlation with carcass traits, specifically smaller carcass REA. Since REA is a contributor to the economic value of a carcass, it could be beneficial for producers to identify this variant in their herds. However, since the allele frequency for the deletion is low the practical utility may be in identification of animals with a genetic potential for smaller REA targeting a specialized market. This may lead to alterations to the number of days to slaughter for cattle based on their *POMC *deletion genotype in order to optimize the fat:muscle ratio of the REA enabling maximization of the yield grade score.

## Methods

### Animal Samples

The University Committee on Animal Care and Supply (UCACS) reviewed and approved both the Beef Research Facility and the tissue collection aspects of this experiment (Protocol numbers 19940033 and 19950068 respectively).

#### Steers

A group of 386 crossbred steers were purchased over the course of 5 days from an auction market near Saskatoon, Saskatchewan in 2005. Auction marts sort animals upon arrival based on sex, size and colour. This presale sorting combined with the fact that we purchased animals over a five-day period makes is unlikely that animals were related. The animals were backgrounded at the University of Saskatchewan (U of S) beef research facility, finished at Pound-Maker Agventures Ltd (Lanigan, SK), and slaughtered at XL Beef (Moose Jaw, SK). The animals received identical backgrounding and finishing diets [17] and were treated identically throughout the experiment.

The backgrounding diet [17] was fed to the steers for an average of 118 days. The following measurements were collected during the backgrounding period: start-of-backgrounding weight (SOB WT), end-of-backgrounding weight (EOB WT), and end-of-backgrounding rib-eye area (EOB REA) and backfat (EOB BF) that were collected by live animal ultrasound. The steers' start-of-finishing weight (SOF WT) was determined on May 2^nd^, and they were shipped to Pound-Maker for finishing on May 3^rd^. During finishing the steers were housed in two pens of approximately 200 animals and both pens were fed a standard finishing diet [17]. End-of-finishing weights (EOF WT) were July 31^st ^and August 1^st^, 2006 at 119 and 120 days on feed, respectively.

The cattle were shipped to XL Beef in Moose Jaw in nine lots between September 5th and September 15th, where they were slaughtered and the following data was collected: hot carcass weight (HCW), average fat, grade fat, carcass rib-eye area (REA), marbling, and cutability. The average fat was calculated by taking the mean value of three fat measurements taken along the 12th rib *longissimus dorsi *muscle, while the grade fat is a measurement taken from the narrowest fat depth over the fourth quadrant of the same muscle. Cutability is an estimate (in percent) of red meat yield.

#### Other Animals and tissue

Allele frequencies were determined using 50 unrelated animals from five major beef cattle breeds (Angus, Charolais, Hereford, Limousin, and Simmental) as well as 20 Holsteins. Tissue samples previously collected from a 19-month old steer included: kidney, testes, liver, rumen wall, skeletal muscle, adrenal gland, brain, small intestine, abomassal muscle, lymph node and thymus (Goodall and Schmutz 2007). Five additional thymus samples were collected. These were from 1 to 2 day old calves that died unexpectedly, samples were collected within minutes of their deaths and immediately placed in RNA*later*(R) (Ambion, Austin, TX).

### DNA/RNA Extraction

The genomic DNA and cDNA from the 19-month old steer were already available [17,18]. Total RNA was isolated from the five-thymus samples following a protocol from Invitrogen (Burlington, ON). Subsequently, cDNA was synthesized using the First Strand cDNA Synthesis Kit from Fermentas (Burlington, ON).

### *POMC *Genotyping

There were three methods used for genotyping *POMC*. For genotyping *POMC* c.288C>T both real time PCR and PCR-RFLP were used. To genotype for the deletion, samples were amplified by PCR and then separated on a 4% agarose gel.

#### Real time PCR of *POMC *c.288C>T

A LightCycler 1.0 (Roche Molecular Biochemicals) was used to perform the real time PCR. The primers and probes are given in Table 3. Total master mix volume was 10.3 μl, consisting of 3 mM of MgCl2, 4 pmol of both forward and reverse primers, 3 pmol of both anchor and sensor probes, 0.7 μl of LightCycler DNA Master HybProbe (Roche Molecular Biochemicals), and 100 ng of DNA. PCR conditions consisted of a denaturation step at 95°C for 10 min, followed by 45 cycles of 95°C for 2 s, 60°C for 10 s, and 72°C for 14 s. The melting program consisted of heating the reaction to 95°C for 0 s and then cooling to 40°C for a period of 120 s, followed by a continuous increasing temperature transition rate of 0.2°C/s until 75°C was reached. In the cooling segment of the program the temperature was lowered to 40°C for 5 s. The donor probe (anchor) was labeled with fluorescein and the receptor probe (sensor) was labeled with LightCycler Red 640 (TIB Molbiol LLC, Adelphia, NJ). The sensor is the acceptor probe for the FRET reaction.

**Table 3:** Primers utilized for POMC genotyping and characterization of variants.

POMC target	Primer	Sequence (5' to 3')
*POMC *c.288C>T	Forward	GATGAGCAGCCGCTGACT
	Reverse	GTCAGCTCCCTCTTGAATTCGAG
	Anchor	GCTTCGGCCGTCGGAATGGT
	Sensor	GCAGCAGCAGCGGAGTTG

12 bp deletion	Forward	CCGTCGGAATGGTAGCA
	Reverse	CGTTGGGGTACACCTTC

cDNA	Forward	GCGACGGAAGAGAACGAAGGA
	Reverse	TGATGGCGTTTTTGAACAGCGTGAC

#### PCR-RFLP of *POMC *c.288C>T

Primers for the PCR-RFLP protocol were as described by Thue and Buchanan [8]. The PCR was performed using a RoboCycler (Stratagene, La Jolla, CA) followed by digestion with *BtsI *endonuclease (New England BioLabs Inc) and electrophoresis on a 2% agarose gel. The undigested PCR product (C allele) is 390 bp, while the digested T allele products are 233 bp and 157 bp in size.

#### Detection of the Twelve bp deletion

Primers to test for the presence/absence of the deletion are presented in Table 3 and the PCR was performed on the LightCycler (Roche Molecular Biochemicals). Total reaction volume was 10 μl, consisting of 3 mM of MgCl2, 6 pmol of both forward and reverse primers, 1.15 M betaine, 0.7 μl of LightCycler DNA Master HybProbe (Roche Molecular Biochemicals) and 100 ng of DNA. The PCR amplification program consisted of denaturation at 95°C for 10 min followed by 37 cycles of 95°C for 2 s, 59°C for 10 s, and 72°C for 14 s. To cool the reaction the temperature was lowered to 40°C for 5 s. PCR products were electrophoresed on a 4% agarose gel. PCR products without the deletion and with the deletion were 206 and 194 bp in size respectively.

### cDNA

PCR was performed using cDNA from the tissues from the 19-month old steer. The forward primer is located within exon 1 while the reverse is within exon 3 (see Table 3).

The total reaction volume used was 15 μl, consisting of 5 pmol per reaction of forward and reverse primers, 200 μM dNTPs, 10× PCR Buffer (Fermentas), 1.5 mM MgCl_2_, 1.3 M betaine, 0.5 U *Taq *DNA polymerase (Invitrogen), and 1 μl of cDNA template. The PCR started with denaturation at 94°C for 2 min, followed by 38 cycles of 94°C for 1 min, 53°C for 45 s, and 72°C for 1 min, and finished with an extension of 72°C for 3 min. PCR products (858 bp) were then electrophorized on a 2% agarose gel.

### Sequencing

PCR products were excised from the agarose gel and extracted using the protocol described in the QIA quick Gel extraction kit (QIAGEN, Mississauga, ON). The purified samples were then sent to the National Research Council of Canada, Plant Biotechnology Institute (Saskatoon, SK) for sequencing. Sequences were analyzed using Sequencher™(Version 4.1. Gene Codes Corporation).

### Statistical analysis

#### Deletion

A Mann Whitney U test compared the production and carcass data of animals that had the deletion to those without the deletion. Only animals that were CT for *POMC *c.288C>T were analyzed (n = 138). Deviations from the Hardy Weinberg Equilibrium were determined. A Chi squared analysis (Statview 5.0) was used to test for significant differences between allele frequencies among the six cattle breeds.

#### *POMC *c.288C>T

When analyzing for an association between *POMC *c.288C>T with production and carcass traits, the 14 animals with the deletion were removed from the data set (n = 372). The mixed procedure of SAS (version 9.1) was then used to analyze the effects of *POMC *c.288C>T on carcass and production traits. Using Proc Mixed, the finishing pen and kill dates were initially taken into account, but were removed subsequently from the model because they were of no significant effect. The following model was used to determine the effect of *POMC *c.288C>T genotype on various traits: Y_ij _= μ + *POMC*_i _+ e_ij _whereYij was the dependent variable for the *i*th observation, μ represents the mean of the dependent variable while *POMC*i is the effect of *POMC *genotype on the dependent variable, and eij represents the random error for each animal observed. Significance was defined at P ≤ 0.05.

## Authors' contributions

HMD was the M.Sc. graduate student who designed a real time PCR for the existing POMC SNP, discovered the deletion, characterized the deletion, genotyped both POMC variants and performed association analysis. FCB previously collected the samples and was the primary graduate supervisor. Both authors were involved in scientific discussions, drafted the manuscript and approved the final version.

## References

[B1] Ingvartsen KL Boisclair YR Leptin and the regulation of food intake, energy homeostasis and immunity with special focus on periparturient ruminants Domest Anim Endocrinol 2001 21 215 250 10.1016/S0739-7240(02)00119-4 11872319

[B2] Pritchard LE Turnbull AV White AJ Pro-opiomelanocortin processing in the hypothalamus: impact on melanocortin signaling and obesity J Endocrinol 2002 172 411 21 10.1677/joe.0.1720411 11874690

[B3] Cool DR Normant E Shen F Chen H Pannel L Zhang Y Loh YP Carboxypeptidase E is a regulated secretory pathway sorting receptor: genetic obliteration leads to endocrine disorders in *Cpefat *mice Cell 1997 88 73 83 10.1016/S0092-8674(00)81860-7 9019408

[B4] Millington GWM The role of proopiomelanocortin (*POMC*) neurons in feeding behaviour Nutr Metab 2007 4 1 16 10.1186/1743-7075-4-18 PMC2018708 17764572

[B5] Raffin-Sanson ML Keyzer Y Bertagna X Proopiomelanocortin, a polypeptide precursor with multiple functions: from physiology to pathological conditions Eur J Endocrinol 2003 149 79 90 10.1530/eje.0.1490079 12887283

[B6] Krude H Biebermann H Lucj W Horn R Brabant G Gruter A Severe-early onset obesity, adrenal insufficiency and red hair pigmentation caused by *POMC *mutations in humans Nat Genet 1998 19 155 157 10.1038/509 9620771

[B7] Giudice EM Cirillo G Santoro N DiUrso L Carbone MT Di Toro R Perrone L Molecular screening of the proopiomelanocortin (*POMC*) gene in Italian obese children: report of three new mutations Int J Obes 2001 25 61 67 10.1038/sj.ijo.0801485 11244459

[B8] Thue TD Buchanan FC Linkage mapping of *POMC *to bovine chromosome 11 Anim Genet 2003 4 146 160 10.1046/j.1365-2052.2003.00965_3.x 12648101

[B9] Buchanan FC Thue TD Yu P Winkelman-Sim DC Single Nucleotide Polymorphisms in the Corticotrophin-releasing Hormone and Pro-opiomelancortin Genes are Associated with Growth and Carcass Yield in Beef Cattle Anim Genet 2005 36 127 131 10.1111/j.1365-2052.2005.01255.x 15771721

[B10] Tung YL Piper SJ Yeung D O'Rahilly S Coll AP A comparative study of the central effects of specific *POMC*-derived melanocortin peptides on food intake and body weight in *POMC *null mice Endocrinology 2006 147 5940 5947 10.1210/en.2006-0866 16959830 PMC2204083

[B11] Denef C Lu J Swinnen E γ-MSH peptides in the pituitary Ann N Y Acad Sci 2003 994 123 132 10.1111/j.1749-6632.2003.tb03171.x 12851307

[B12] PredictProtein http://www.predictprotein.org

[B13] Fernandez CJ Warren G In vitro synthesis of sulfated glycosaminoglycans coupled to inter-compartmental golgi transport J Biochem Mol Biol 1998 723 19030 19039 10.1074/jbc.273.30.19030 9668084

[B14] Yaswen L Diehl N Brennan MB Hochgeschwender U Obesity in the mouse model of pro-opiomelanocortin deficiency responds to peripheral melanocortin Nature 1999 5 1066 1070 10.1038/12506 10470087

[B15] Chamary JV Hurst LD The price of silent mutations Sci Am 2009 300 46 53 10.1038/scientificamerican0609-46 19485088

[B16] Kudla G Lipinski L Caffin F Helwak A Zylicz M High guanine and cytosine content increases mRNA levels in mammalian cells PLoS Biol 2006 4 933 942 10.1371/journal.pbio.0040180 PMC1463026 16700628

[B17] Pugh K An evaluation of the *corticotrophin-releasing hormone *and *leptin *gene SNPs relative to cattle behaviour M.Sc. thesis 2007 University of Saskatchewan, Animal and Poultry Science

[B18] Goodall JJ Schmutz SM IGF2 gene characterization and association with rib eye area in beef cattle Anim Genet 2007 38 154 161 10.1111/j.1365-2052.2007.01576.x 17403010

